# The effect of perceived social support on postpartum stress: the mediating roles of marital satisfaction and maternal postnatal attachment

**DOI:** 10.1186/s12905-023-02593-9

**Published:** 2023-09-11

**Authors:** Yanchi Wang, Jian Gu, Feng Zhang, Xujuan Xu

**Affiliations:** 1 Medical School of Nantong University, Nantong, Jiangsu China; 2 Department of Nursing, Affiliated Hospital of Nantong University, Nantong, 226001 Jiangsu China; 3 Affiliated Nantong Hospital of Shanghai University (The Sixth People’s Hospital of Nantong), Nantong, Jiangsu China; 4 Department of Epidemiology and Medical Statistics, School of Public Health, Nantong University, Nantong, Jiangsu China

**Keywords:** Postpartum stress, Perceived social support, Marital satisfaction, Maternal and infant attachment, Mediating effect

## Abstract

**Background:**

Multiple factors may be responsible for the development of postpartum stress, including perceived social support, marital satisfaction, and maternal postnatal attachment. However, the underlying mediation mechanisms remain unclear. This study examined the complex relationships between perceived social support and postpartum stress among Chinese women.

**Methods:**

A convenience sample comprising 406 postpartum women was recruited from six hospitals in Nantong, Jiangsu Province, China. The participants completed general survey questionnaires and were evaluated using the Maternal Postpartum Stress Scale, the Perceived Social Support Scale, the Maternal Postnatal Attachment Scale, and the Marital Satisfaction Scale. Furthermore, we evaluated the relationship between postpartum stress and the various influencing factors by performing a multiple linear regression analysis. The potential mediating roles of marital satisfaction and maternal and infant attachment in the association between perceived social support and postpartum stress were explored by performing a mediation analysis.

**Results:**

According to the multivariate regression analysis, perceived social support, marital satisfaction, and maternal postnatal attachment contributed to postpartum stress levels (*P* < 0.05). The mediation analysis revealed that marital satisfaction and maternal postnatal attachment played parallel mediating roles in the association between perceived social support and postpartum stress, and the mediating effect of marital satisfaction was − 0.1125 (95% confidence interval [CI]: -0.1784 to -0.0520), accounting for 33.20% of the total effect, and the mediating effect of maternal postnatal attachment was − 0.0847 (95% CI: -0.1304 to -0.0438), accounting for 25.00% of the total effect.

**Conclusion:**

Our study revealed that perceived social support could influence postpartum stress not only through direct effect (41.80% of the total effect), but also through the indirect effect (mediation effect) of marital satisfaction and maternal postnatal attachment (58.20% of the total effect), suggesting that improving postpartum women’s social support, enhancing maternal and infant attachment, and improving their marital satisfaction could help lower postpartum stress.

## Introduction

Postpartum stress is conceptualised as a negative psychological response to the numerous obligations associated with raising children, and its presence is the rule rather than the exception [1]. Increasing evidence has reported that postpartum stress is one of the most common psychiatric disorders in postpartum women and is associated with a high burden of mental illness [2–4]. Additionally, postpartum stress is more common than postpartum depression, affecting a considerable proportion of mothers [5]. Generally, the period after childbirth is when the mother is most vulnerable to emotional changes [6]. If postpartum women cannot actively handle postpartum stress, they may develop self-doubt and have deviations in their self-evaluation. This might cause postpartum stress to progress into severe postpartum mental diseases, such as postpartum depression [7, 8]. This might result in suicidal thoughts or thoughts of causing harm to the infant [9]. Besides, it could also impact the couple’s relationships, the familial environment, and, particularly, the infant’s welfare [10]. Thus, investigating the factors and underlying mechanisms contributing to postpartum stress and employing preventive measures might help promote maternal physical and mental health.

Several factors, particularly perceived social support, might contribute to postpartum stress. A systematic review reported five key themes that contribute to postpartum stress, namely social support, women’s experience with health care, social norms and expectations, factors that influence coping, and maternal and child health [11]. Low perceived social support might predict postpartum stress [12]. A study comprising 859 postpartum women within 6 weeks after delivery in demonstrated that decreased social support was a significant predictor for postpartum stress [13]. In a regional-epidemiological sample of 306 German women, peripartum depressive, anxiety, and stress symptoms were lower in women with higher perceived social support [14]. Besides, in a Canadian study including 3,388 women who measure stress, anxiety and social support across 4 time points from pregnancy to 12 months postpartum, and results suggested that increases in both partner and family support may decrease stress and anxiety during pregnancy and the postpartum [15]. Thus, it is possible that tangible social support could lower postpartum stress [16, 17]. Although the relationship between social support and postpartum stress is well established, the underlying mechanisms mediating the impact of perceived social support on postpartum stress are yet to be elucidated.

Besides perceived social support, marital satisfaction and maternal postnatal attachment are two significant factors that contribute to postpartum stress [18, 19]. Reportedly, marital satisfaction declines after the birth of a couple’s first child [20]. Marital dissatisfaction is associated with an increased risk for postpartum stress [21], and those who are not satisfied with their marriage appeared to be associated with more stress and anxiety [22].Besides, with regard to the associated factors influencing parenting stress, anxiety, depression, and marital satisfaction may contribute to parenting stress [23].

During the postpartum period, social support is crucial, and the partner is typically the main source of that support. Poor emotional well-being and lower marital satisfaction are associated with a partner’s lack of support [24]. Findings have shown that social support has a direct positive effect on marital satisfaction [25]. Besides, the association between perceived social support and postpartum stress might be mediated by marital satisfaction, a study of 1,585 new mothers in Korea reported that the moderating effect of social support on postpartum psychological distress is contingent on levels of marital quality [26]. Less postpartum stress and fewer declines in marital functioning were observed in mothers who received greater social support [27]. However, the underlying mechanism and the degree that marital satisfaction mediating the association between perceived social support and postpartum stress are yet to be further elucidated.

Maternal psychological distress is associated with postpartum mother-infant bonding problems [28], and maternal postnatal attachment is significantly negatively associated with postpartum stress [29, 30]. In a Polish sample including 150 women, it is reported that maternal stress are significantly associated with the maternal-infant bonding process in the early postpartum period [31]. In addition, adequate social support tends to strengthen maternal postnatal attachment since it is a psychologically satisfying state and maternal antenatal attachment and social support significantly predicted postnatal maternal-infant attachment [32].Therefore, it is possible that maternal postnatal attachment might mediate the association between perceived social support and postpartum stress. According to some studies, a negative correlation was observed between maternal postnatal attachment and postpartum stress, whereas social support may positively influence maternal postnatal attachment [32, 33]. However, the underlying mechanism and the extent that maternal postnatal attachment mediating the association between perceived social support and postpartum stress are yet to be further elucidated.

Thus, previous studies have separately reported that perceived social support, marital satisfaction and maternal postnatal attachment may affect postpartum stress, and it is possible that marital satisfaction/maternal postnatal attachment might mediate the association between perceived social support and postpartum stress. However, no study has reported chain mediations between both of the three factors (perceived social support, marital satisfaction and maternal postnatal attachment) and postpartum stress. Therefore, this study hypothesised that marital satisfaction and maternal postnatal attachment might serve as parallel mediators for the association between perceived social support and postpartum stress. This study aimed to investigate the following: (1) the level of postpartum stress in Chinese postpartum women and (2) the parallel mediation mechanism, wherein marital satisfaction and maternal postnatal attachment might mediate the association between perceived social support and postpartum stress. The findings of the study will deepen our understanding of the mechanism underlying postpartum stress, which will also help us develop effective prevention and interventional strategies in the future.

## Materials and methods

### Participants

The inclusion criteria were as follows: (1) those with a maternal age of ≥ 18 years; (2) newborns with no malformations or serious complications; (3) postpartum women who were willing to cooperate during the survey, communicate normally, understand the contents of the questionnaire, and fill in the questionnaire independently; (4) those who could provide written informed consent. The exclusion criteria were as follows: (1) mothers who were unmarried, had a preterm delivery, or gave multiple births; (2) those with comorbid psychosomatic diseases, such as chronic urticaria, neurodermatitis, hyperthyroidism, migraine, muscle pain, rheumatoid arthritis, sleep disorders, and malignant tumours; (3) those with mental illnesses other than depression and anxiety, those with major diseases of the heart, liver, and kidney, and those with severe complications during pregnancy; (4) those with low intelligence and inability to understand the contents of the questionnaire; (5) postpartum women with poor compliance and who were unwilling to cooperate. A total of 425 postpartum women were surveyed, of which three dropped out, eight had missing data, and eight provided invalid answers. Finally, 406 valid questionnaires were eventually returned with a survey response rate of 95.53%.

### Data collection

Primary data which is directly collected from the postpartum women through face-to-face questionnaire were collected from August to November 2022. Postpartum women who underwent physical examination 6‒8 weeks after delivery at six hospitals in Nantong, Jiangsu Province, China, were selected as the research participants using the convenience sampling method. The detailed method of selection is as follows: Introduce yourself as a researcher and briefly explain the purpose and significance of the study to postpartum women who met the inclusion criteria at the postpartum clinics. Explain the voluntary nature of participation and the importance of obtaining informed consent. Clarify that participants have the right to decline or withdraw from the study at any time without any consequences. Provide a consent form or document that outlines the study details, including the purpose, data collection methods, potential risks or benefits, confidentiality measures, and contact information for any questions or concerns. Describe the data collection process and the specific methods being used. In this case, mention that a questionnaire will be administered to gather information related to their postpartum experience. Highlight that their responses will be anonymous and confidential, and that their personal identification will not be linked to the questionnaire. Once participants have agreed to participate, provide them with the questionnaire. Explain any specific instructions for completing it, or any additional notes or comments they can provide. The Ethics Committee of the Affiliated Hospital of Nantong University (approval number: 2022-K50-01) approved this study.

### Measurement

#### Sociodemographic characteristics

The research team members designed the questionnaire regarding the general characteristics of postpartum women. The main variables comprised the following: (1) basic characteristics of the postpartum women: age, body mass index (BMI), education (maternal), education (husband), family monthly income, years of marriage, and medical insurance; (2) perinatal conditions: mode of delivery, abortion history, parity, pregnancy complications, assisted reproduction, and infant caretaker; (3) basic information of the newborns: birth weight and feeding.

#### Maternal Postpartum stress scale (MPSS)

MPSS [34] is a reliable and valid instrument measuring self-reported postpartum stress among postpartum women. This scale comprises 22 items measuring three subscales of postpartum stress, namely personal needs and fatigue (nine items), infant nurturing (seven items), and body changes and sexuality (six items). Each item of the scale is scored on a 5-point Likert scoring ranging from 0 (not at all) to 4 (completely). A higher MPSS total score indicates a higher level of postpartum stress. Cronbach’s α coefficient of internal consistency of the Chinese-version MPSS verified by the researchers was 0.940.

#### Perceived Social Support Scale (PSSS)

The PSSS was used to measure social support [35]. The PSSS is a 12-item instrument that assesses support from family, friends, relatives, and colleagues. Each item is rated on a 7-point Likert scale ranging from 1 (strong disagreement) to 7 (strong agreement). The total score ranges from 12 to 84, with higher scores indicating a higher perceived social support. A score between 12 and 36 indicates low support, between 37 and 60 indicates medium support, and between 61 and 84 indicates high support. Cronbach’s α for PSSS in the current study was 0.914 [36].

#### Marital satisfaction scale (MSS)

The MSS-shortened version [37] comprises 10 items measuring marital satisfaction. Each item is scored on a 5-point Likert scale, with the answers ranging from “5 = I quite agree with” to “1 = I quite disagree with”. Question numbers Q1, Q3, Q5, Q8, and Q9 are negative items and need reversing. The total score of this questionnaire ranges from 10 to 50. A higher score indicates higher marital satisfaction.

#### Maternal postnatal attachment scale (MPAS)

MPAS [38] comprises 19 items, including attachment quality, interactive pleasure, and three non-hostile subscales. Each item is scored on a scale of 1 to 5 points, with the total score ranging from 19 to 95 points. A higher score indicates a higher attachment level. 

### Statistical methods

The general characteristics were presented using descriptive analyses (e.g., mean, standard deviation [SD], frequency, constituent ratio, etc.). Considering the data of MPSS scores does not conform to the normal distribution, so before doing statistical analyses, we normalized the scores of MPSS by rank-based inverse-normal transformation (INT). After INT, the score of MPSS conforms to the normal distribution, thus Student’s t-tests and one-way analysis of variance were used to assess the differences in MPSS scores (after INT) for each group’s demographic characteristics, while multiple linear regression analysis was used to assess the association between each scale and postpartum stress levels (MPSS scores after INT).

The correlation between variables was analysed using Pearson’s correlation analysis. Parallel mediation modelling analyses were used to investigate the association between perceived social support and marital satisfaction and maternal postnatal attachment and postpartum stress. The bootstrap method (5000 times) was used to examine the mediating effects, providing 95% confidence intervals (CIs). SPSS (version 25.0, IBM Corp) and SPSS PROCESS macro (version 3.3) were used to perform statistical analyses. Variables used in multiple linear regression analysis and parallel mediation modelling analyses including scores of MPSS, PSSS, MSS and MPAS.R version 3.6.2 was used to draw all figures. Type I error was set at *P* < 0.05 (two-sided) for all statistical analyses.

## Results

### Characteristics of the study population

Table 1 presents the data on the general characteristics, including age, education (maternal), education (husband), family monthly income, medical insurance, feeding, mode of delivery, years of marriage, abortion history, parity, assisted reproduction, BMI, complications of pregnancy, birth weight, infant caretaker, and MPSS values of the 406 participants included in this study. The MPSS scores (mean ± SD) in terms of each group’s demographic characteristics were comparable since no significant differences were observed (*P* > 0.05).

**Table 1:** Characteristics of the subjects enrolled in this study

Variables		Scores of MPSS		*P*		Variables		Scores of MPSS		*P*
		N (%)	Mean ± SD					N (%)	Mean ± SD	
Age	< 30 years	228 (56.16)	16.05 ± 12.71	0.120		Years of	≤ 5 years	324 (79.80)	16.79 ± 12.56	0.919
	≥ 30 years	178 (43.84)	17.97 ± 12.34			marriage	> 5 years	82 (20.20)	17.30 ± 12.69	
Education	Below bachelor	158 (38.92)	15.99 ± 12.81	0.109		Abortion	No	274 (67.49)	16.58 ± 12.66	0.460
(maternal)	Bachelor or above	248 (61.08)	17.46 ± 12.41			history	Yes	132 (32.51)	17.55 ± 12.40	
Education	Below bachelor	171 (42.12)	17.32 ± 12.83	0.753		Parity	Primipara	317 (78.08)	17.06 ± 12.36	0.355
(husband)	Bachelor or above	235 (57.88)	16.58 ± 12.40				Multipara	89 (21.92)	16.29 ± 13.37	
Family monthly income (CNY ¥)	< 5000	15 (3.70)	18.87 ± 14.73	0.765		Assisted	No	345 (84.98)	16.64 ± 12.63	0.422
	5000–10,000	141 (34.73)	16.28 ± 12.74			reproduction	Yes	61 (15.02)	18.31 ± 12.21	
	10,001–20,000	183 (45.07)	17.49 ± 12.66			BMI (kg/m^2^)	Normal	245 (60.34)	16.83 ± 12.47	0.891
	> 20,000	67 (16.50)	16.10 ± 11.61				Overweight	98 (24.14)	16.34 ± 12.10	
Medical	No	56 (13.79)	16.43 ± 11.02	0.858			Obesity	63 (15.52)	17.98 ± 13.75	
insurance	Yes	350 (86.21)	16.97 ± 12.81			Complications	No	281 (69.21)	17.04 ± 12.90	0.680
Feeding	Breast-feeding	163 (40.15)	16.58 ± 13.03	0.396		of pregnancy	Yes	125 (30.79)	16.57 ± 11.85	
	Mixed-feeding	204 (50.25)	17.46 ± 12.39			Birth weight	< 3000 g	69 (17.00)	18.03 ± 11.32	0.420
	Artificial-feeding	39 (9.60)	15.21 ± 11.65				3000-4000 g	296 (72.90)	16.43 ± 12.80	
Mode of delivery	Vaginal	179 (44.09)	15.84 ± 12.04	0.363			> 4000 g	41 (10.10)	18.32 ± 12.96	
	Vaginal (lateral episiotomy)	71 (17.49)	17.01 ± 14.08			Infant	Only oneself	110 (27.09)	17.34 ± 13.66	0.587
	Cesarean section	156 (38.42)	18.04 ± 12.43			caretaker	Involving others	296 (72.91)	16.73 ± 12.16	

**Table 2:** Results of linear regression analysis on postpartum stress

Variables	β	SE	95% CI		*P*
			Lower	Upper	
**A: univariate**					
Perceived Social Support	-0.026	0.004	-0.033	-0.018	< 0.001
Marital Satisfaction	-0.050	0.006	-0.063	-0.038	< 0.001
Maternal Postnatal Attachment	-0.063	0.008	-0.080	-0.047	< 0.001
**B: multivariate**					
Perceived Social Support	-0.009	0.004	-0.018	0.000	0.037
Marital Satisfaction	-0.030	0.007	-0.045	-0.016	< 0.001
Maternal Postnatal Attachment	-0.042	0.009	-0.059	-0.025	< 0.001

### Levels of postpartum stress across the study sample

**Fig. 1:**
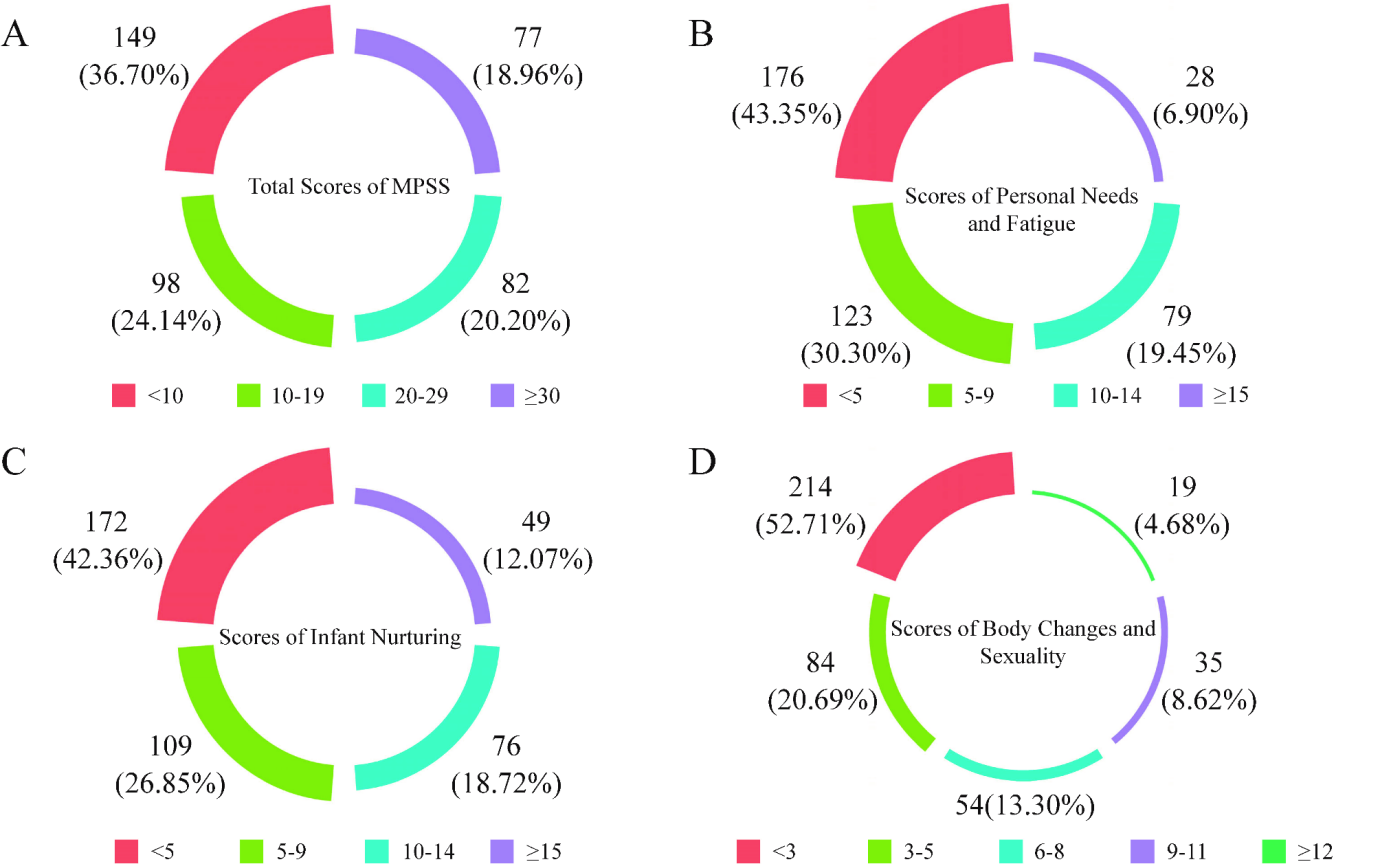
Frequency of the participants in each item of the Maternal Postpartum Stress Scale (MPSS) and subscale groups. (**A**) Total MPSS scores; (**B**) Personal needs and fatigue scores; (**C**) Infant nurturing scores; (**D**) Body change and sexuality scores

Of the 406 participants, the postpartum stress scores of 149 (36.70%) postpartum women were < 10 points, of 98 (24.14%) postpartum women were 10–19 points, of 82 (20.20%) postpartum women were 20–29 points, and of 77 (18.96%) postpartum women were ≥ 30 points. The postpartum stress varies based on personal needs and fatigue, infant nurturing, body changes and sexuality. The median MPSS score was 15, while the median scores of the three MPSS subscales were 5 (personal needs and fatigue), 6 (infant nurturing), and 3 (body changes and sexuality), respectively. Details of the number of participants in each score group are presented in Fig. 1.

### Associations among perceived social support, marital satisfaction, maternal postnatal attachment, and postpartum stress

Perceived social support (β: -0.026, 95% CI: -0.033 to -0.018, *P* < 0.001), marital satisfaction (β: -0.050, 95% CI: -0.063 to -0.038, *P* < 0.001), and maternal postnatal attachment (β: -0.063, 95% CI: -0.080 to -0.047, *P* < 0.001) were significantly associated with altered postpartum stress levels (Table 2 A). Multivariate regression analysis was then used for the above three factors to identify the independent factors contributing to postpartum stress levels. As presented in Table 2B, perceived social support (β: -0.009, 95% CI: -0.018 to 0.000), marital satisfaction (β: -0.030, 95% CI: -0.045 to -0.016), and maternal postnatal attachment (β: -0.042, 95% CI: -0.059 to -0.025) collectively had a *P*-value of < 0.05, indicating higher perceived social support, higher marital satisfaction, and higher maternal postnatal attachment were negatively significantly associated with lower postpartum stress levels.

### Correlation analysis

**Fig. 2:**
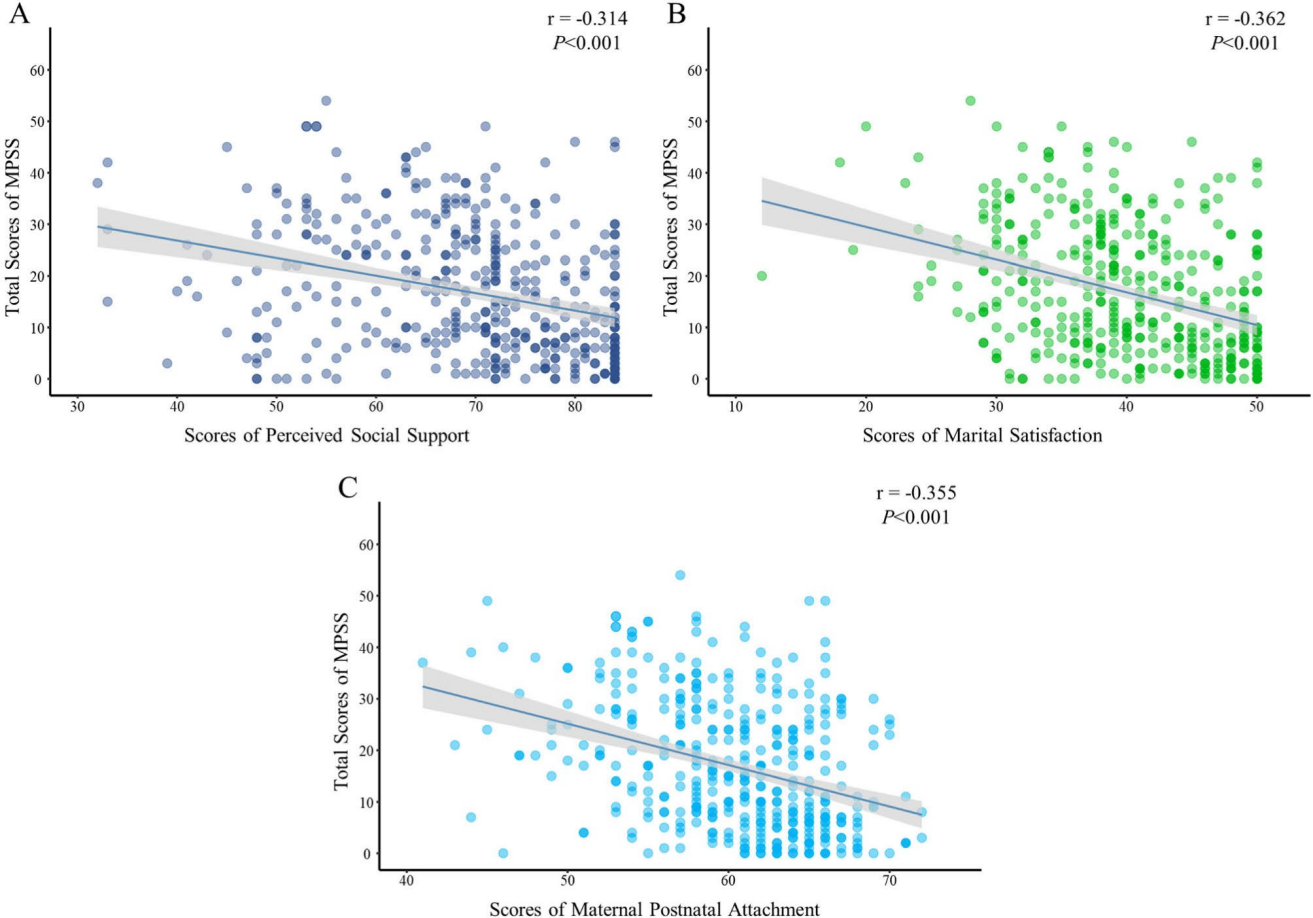
Correlations between perceived social support, marital satisfaction, maternal postnatal attachment, and postpartum stress. (**A**) Correlations between perceived social support and postpartum stress; (**B**) correlations between marital satisfaction and postpartum stress; (**C**) correlations between maternal postnatal attachment and postpartum stress

As presented in Table 3, the average postpartum stress score was 16.89 ± 12.57. A significant and negative correlation was observed between postpartum stress and marital satisfaction (r = -0.362, *P* < 0.01). Besides, a significant negative correlation was observed between postpartum stress and perceived social support (r = -0.314, *P* < 0.01) and maternal postnatal attachment (r = -0.355, *P* < 0.01). The correlation between postpartum stress and the above factors is presented in Fig. 2.

**Table 3:** Mean, standard deviation (SD), and correlations for study variables (N = 406)

Variables	Postpartum Stress	Perceived Social Support	Marital Satisfaction	Maternal Postnatal Attachment
Postpartum Stress	1			
Perceived Social Support	-0.314^**^	1		
Marital Satisfaction	-0.362^**^	0.506^**^	1	
Maternal Postnatal Attachment	-0.355^**^	0.340^**^	0.385^**^	1
Mean	16.89	69.44	40.00	60.40
Standard deviation (SD)	12.57	11.62	7.16	5.54

**P < 0.01 (two-tailed test)

### Mediation analysis

The correlation analysis reported a significant association between perceived social support and postpartum stress, wherein higher perceived social support was associated with lower postpartum stress. However, after adding the mediators to the model, this effect significantly declined, and the direct effect was − 0.1416 (95% CI: -0.2536 to -0.0297, *P* < 0.05), accounting for only 41.80% of the total effect. Thus, the direct effect accounted for 41.80% on the association between perceived social support and postpartum stress.

**Fig. 3:**
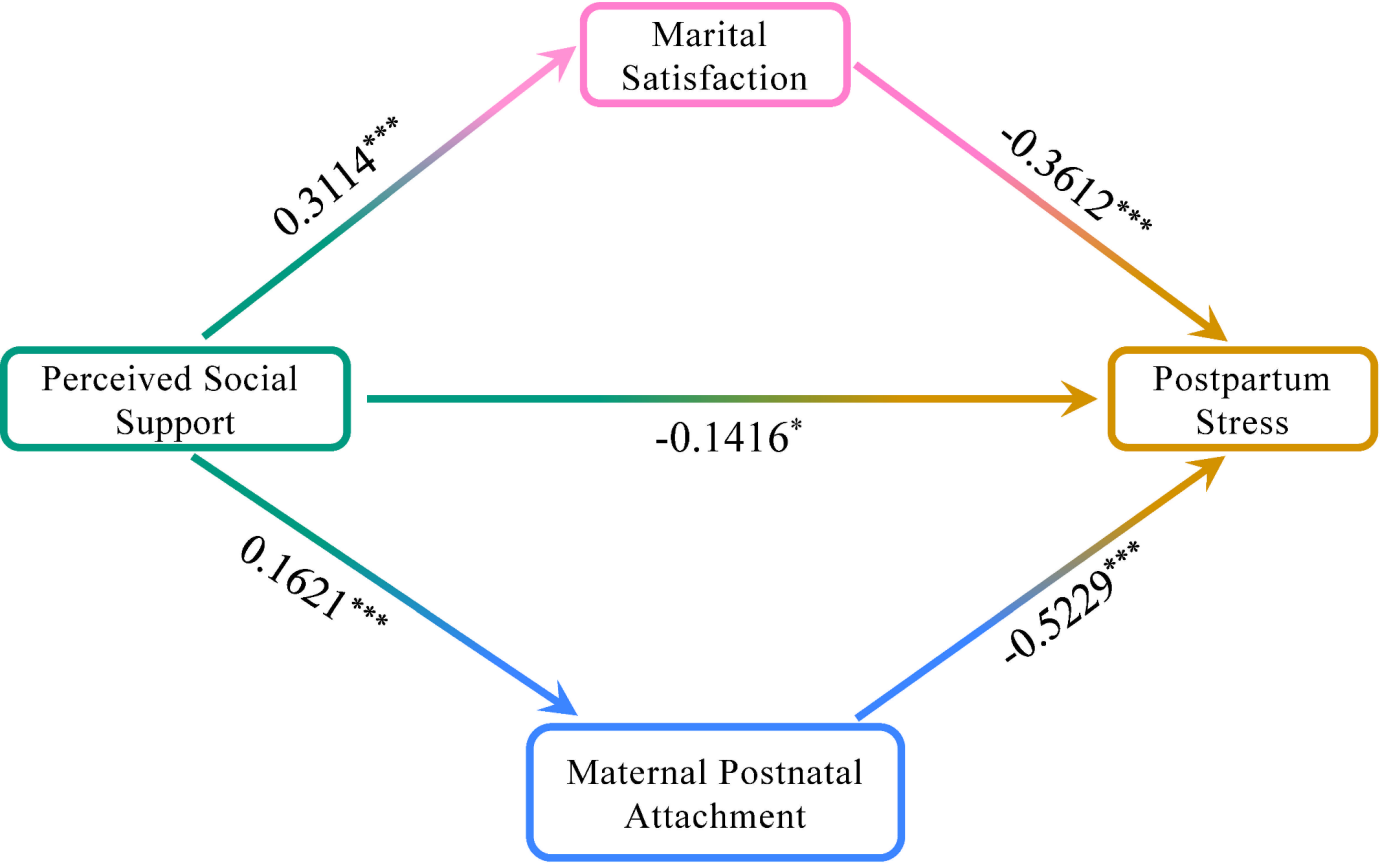
The parallel mediation model of perceived social support, marital satisfaction, maternal postnatal attachment, and postpartum stress. *** *P* < 0.001, * *P* < 0.05

We then identified the parallel mediating effects of marital satisfaction and maternal postnatal attachment on the association between perceived social support and postpartum stress (Fig. 3**)**. The overall mediating effect of marital satisfaction and maternal postnatal attachment was − 0.1972 (95% CI: -0.2663 to -0.1329), accounting for 58.20% of the total effect. Specifically, the mediating effect of marital satisfaction was − 0.1125 (95% CI: -0.1784 to -0.0520), accounting for 33.20% of the total effect, and the mediating effect of maternal postnatal attachment was − 0.0847 (95% CI -0.1304 to -0.0438), accounting for 25.00% of the total effect (Table 4). Thus, the indirect effect (mediation effect) of marital satisfaction and maternal postnatal attachment accounted for 58.20% on the association between perceived social support and postpartum stress.

**Table 4:** The parallel mediating effect of marital satisfaction and maternal postnatal attachment on the relationship between perceived social support and postpartum stress (incompletely standardized indirect effect(s) of X on Y)

Model Pathways	Effect	Boot SE	95% CI		Relative Mediation
			BootLL CI	BootUL CI	Effect %
Direct effect	-0.1416	0.0569	-0.2536	-0.0297	41.80%
perceived social support →marital satisfaction →postpartum stress	-0.1125	0.0319	-0.1784	-0.0520	33.20%
perceived social support →maternal postnatal attachment → postpartum stress	-0.0847	0.0222	-0.1304	-0.0438	25.00%
Total mediation effect	-0.1972	0.0338	-0.2663	-0.1329	58.20%
Total effect	-0.3389	0.0511	-0.4393	-0.2385	100.00%

## Discussion

Postpartum stress is a challenge for postpartum women. This study investigated the postpartum stress level and evaluated the complex associations between perceived social support, marital satisfaction, maternal postnatal attachment, and postpartum stress using the parallel mediation modelling analysis. The findings of the study might deepen our understanding of the underlying mechanisms between perceived social support and postpartum stress and provide new evidence for effective interventional and prevention strategies targeting postpartum stress in the future.

The study results revealed that the mean of the postpartum stress summated scores was 16.89 (SD = 12.57), with more than one-third of the women having a postpartum stress score of < 10 points, less than one-fourth of the women having a postpartum stress score of 10–19 points, more than one-fifth of the women having a postpartum stress score of 20–29 points, and 18.96% of the women having a postpartum stress score of ≥ 30 points. The effects of postpartum stress varied based on personal needs and fatigue, infant nurturing, body changes and sexuality. A study conducted in Taiwan (China) evaluated maternal role attainment, lack of social support, and negative body changes as the three aspects of postpartum stress [39]. Several mothers do not transition well to their parental roles [40], and some do not breastfeed for work or health reasons [41–43]; therefore, the attachment between the mother and the infant lacks strength. Negative body changes can lessen the level of attraction between partners, thereby affecting intimacy [44]. Partner’s emotions were significantly associated with postpartum stress [45]. An increased level of stress was observed in women 6 weeks after delivery, and a gradual decline was observed in the marital relationship from pregnancy to 1 year postpartum [46]. However, partners with cognitive empathy can alleviate marital conflict, increase marital satisfaction, and lower postpartum stress [47]. A decreased infant sleep time and a tense relationship between postpartum women and their mothers-in-law were associated with postpartum depression [48]. Satisfaction with the infant’s sex, the fathers’ adaptation, and family resources also contribute to postpartum stress [49].

The study results revealed that characteristics of the study sample, including age and education level, were not significantly associated with postpartum stress. On the one hand, the population lacked heterogeneity, and on the other hand, the characteristics of the postpartum women could not predict postpartum stress. Previous studies have reported that increased maternal postnatal attachment, marital satisfaction, and social support were associated with decreased postpartum stress from the first week to the fourth week after delivery [50]. This study reported that perceived social support was significantly associated with postpartum stress. The result emphasised the significance of prefill interventions for postpartum women, particularly for those with postpartum stress. This study could accurately lower postpartum stress in women rather than only focusing on the adverse consequences of postpartum stress. Marital satisfaction and maternal postnatal attachment played parallel mediating roles in the association between perceived social support and postpartum stress, and the mediating effect of marital satisfaction was more significant than maternal postnatal attachment.

The results have reported that marital satisfaction partially mediates the association between perceived social support and postpartum stress. Higher marital satisfaction was associated with a higher quality of life, thereby making postpartum women experience increased social support, resulting in decreased postpartum stress [51]. Mothers who perceived stronger social support from their partners experienced lower postpartum emotional distress [52]. The transition to parenthood and increased postpartum demands result in decreased marital satisfaction and perceived social support, thereby increasing the risk of postpartum stress [53]. Low marital satisfaction could result in social loneliness, thereby mediating the relationship between perceived social support and postpartum stress [54].

Additionally, the findings of this study reported that maternal postnatal attachment partially mediated the relationship between perceived social support and postpartum stress. A significant negative correlation was observed between maternal-infant bonding and postpartum stress; however, a positive correlation was observed between maternal-infant bonding and partner support with the infant and social support. Maternal postnatal attachment partially mediated the effects of perceived social support on postpartum stress, which was consistent with findings in studies conducted among women having their first child in Pennsylvania, United States of America [30]. Postpartum women with decreased maternal and infant attachment experience increased postpartum stress and lesser partner support [55]. Promoting a positive maternal-infant attachment could alleviate postpartum stress [56–58]. Breastfeeding and infant sleep can directly affect maternal and infant attachment, thereby impacting postpartum stress [59]. Breastfeeding guidance and infant rearing should be emphasised with adequate social support.

The findings of this study can be used to develop interventions to prevent postpartum stress if further confirmed in a longitudinal study. Considering the significant roles of marital satisfaction and maternal postnatal attachment in mediating the association between perceived social support and postpartum stress, future interventional strategies might aim to improve the postpartum mother-infant connection and postpartum marital intimacy. Thus, enhancing social support for postpartum women through concern from the family and positive workplace interactions might be a key method in preventing postpartum stress. The typical measures include organising support groups, conducting mindfulness-based courses, implementing stress management programs, and conflict-resolution training. Additionally, given the significant influence that marital relationships and mother-infant relationships have on postpartum stress, infant nurturing consultation and marital counselling should also be considered to assist postpartum women in resolving their issues with their partners and infant before moving on to more severe issues.

Our study has several limitations. First, although parallel mediation may extend the typical mediation analysis by testing several mediators within a single model to estimate the direct and indirect effects of the variables simultaneously, we could not reach such a conclusion that the parallel mediation modelling analysis can completely test that complexity, and other analysis are warranted to test and validate this complexity. Second, there are many factors that may influence postpartum stress, and that we could not exclude the possibility that other factors may also act as mediators on the effect of perceived social support on postpartum stress, further studies are warranted to extend our findings. Third, although convenience sampling method provides the advantage of expedited results, especially including the ability to distribute surveys widely in a timely manner; however, this non- randomization method is prone to suffer from the issue of selection bias, which may prevent the generalizability of the result; further studies are warranted to validate our results. Fourth, although the aim of this study is to examine the underlying mechanism and the degree that marital satisfaction and maternal postnatal attachment mediating the association between perceived social support and postpartum stress. Considering the subscale ‘Family Support’ of the Perceived Social Support Scale includes supports from husband, although husband accounts for only a small part of ‘Family Support’, we could not exclude the possibility that the associated direction might also be “Marital Satisfaction → Perceived Social Support”, further studies are warranted to evaluate this. Last, although the MPSS scores in terms of each group of the participants’ background information were comparable (*P* > 0.05), we could not exclude the possibility that these variables related to participants’ background information may be related to each outcome of our study, further analyses including variables of MPSS, PSSS, MSS and MPAS, as well as participants’ background information may extend our findings.

## Conclusion

Our study revealed that perceived social support could influence postpartum stress not only through direct effect (41.80% of the total effect), but also through the indirect effect (mediation effect) of marital satisfaction and maternal postnatal attachment (58.20% of the total effect). Therefore, increasing the social support given to postpartum women, enhancing maternal and infant attachment, and improving their marital satisfaction could help lower their postpartum stress.

## Data Availability

The datasets used and/or analysed during the current study available from the corresponding author on reasonable request.
